# Liver transplantation is the major determinant of ≥10-year survival in patients with hepatocellular carcinoma

**DOI:** 10.1097/HC9.0000000000000951

**Published:** 2026-04-24

**Authors:** Mohammad Saeid Rezaee-Zavareh, Joseph C. Ahn, Hyunseok Kim, Michael Luu, Walid Ayoub, Alexander Kuo, Hirsh Trivedi, Yun Wang, Aarshi Vipani, Tsuyoshi Todo, Georgios Voidonikolas, Justin A. Steggerda, Steven A. Wisel, Todd V. Brennan, Cristina Ferrone, Irene K. Kim, Kambiz Kosari, Nicholas Nissen, Neehar D. Parikh, Amit G. Singal, Ju Dong Yang

**Affiliations:** 1 Middle East Liver Diseases (MELD) Center, Tehran, Iran; 2 Division of Gastroenterology and Hepatology, Mayo Clinic, Rochester, Minnesota, USA; 3 Karsh Division of Gastroenterology and Hepatology, Cedars-Sinai Medical Center, Los Angeles, California, USA; 4 Comprehensive Transplant Center, Cedars-Sinai Medical Center, Los Angeles, California, USA; 5 Biostatistics and Bioinformatics Research Center, Cedars-Sinai Medical Center, Los Angeles, California, USA; 6 Department of Surgery, Cedars-Sinai Medical Center, Los Angeles, California, USA; 7 Division of Gastroenterology, University of Michigan, Ann Arbor, Michigan, USA; 8 Harold C. Simmons Comprehensive Cancer Center, UT Southwestern Medical Center, Dallas, Texas, USA; 9 Division of Digestive and Liver Diseases, UT Southwestern Medical Center, Dallas, Texas, USA; 10 Samuel Oschin Comprehensive Cancer Institute, Cedars-Sinai Medical Center, Los Angeles, California, USA

**Keywords:** ablation, liver cancer, long-term survival, National Cancer Database, surgical resection, transplantation

## Abstract

**Background:**

Hepatocellular carcinoma (HCC) has a poor long-term prognosis due to high recurrence and cirrhosis-related mortality, even after potentially curative treatments such as liver transplantation (LT), surgical resection, or ablation. This study aimed to identify factors associated with ≥10-year survival in HCC patients.

**Methods:**

A retrospective cohort study was conducted among HCC patients diagnosed between 2004 and 2022 using the National Cancer Database. Multivariable Cox regression was used to identify predictors of overall survival, and logistic regression was used to identify predictors of ≥10-year survival.

**Results:**

Among 249,600 HCC patients, 177,585 (71.2%) died within 5 years, 8613 (3.5%) died at 5–10 years, 54,988 (22.0%) were alive with <10 years of follow-up, and 8219 (3.3%) survived ≥10 years. LT, resection, and ablation were performed in 6.6%, 9.3%, and 11% of patients, respectively. Compared with ablation as the reference group, LT [adjusted odds ratio (aOR) 11.96, 95% confidence interval (CI): 11.27–13.29] and resection (aOR: 2.83, 95% CI: 2.57–3.08) increased the odds of ≥10-year survival, while non-curative treatments reduced the odds compared with ablation (aOR: 0.50, 95% CI: 0.47–0.55). Cox regression results were consistent with the logistic model, confirming the association. Decision tree analysis confirmed LT as the dominant determinant of long-term survival. Black individuals were associated with lower odds of ≥10-year survival (aOR: 0.88, 95% CI: 0.820–0.96) and decreased likelihood of receiving LT (aOR: 0.73, 95% CI: 0.55–0.96).

**Conclusions:**

LT offers the best chance of ≥10-year survival in HCC. Ensuring equitable access is essential, especially for Black patients who have lower transplant rates and worse outcomes.

## Graphical abstract

**Figure:**
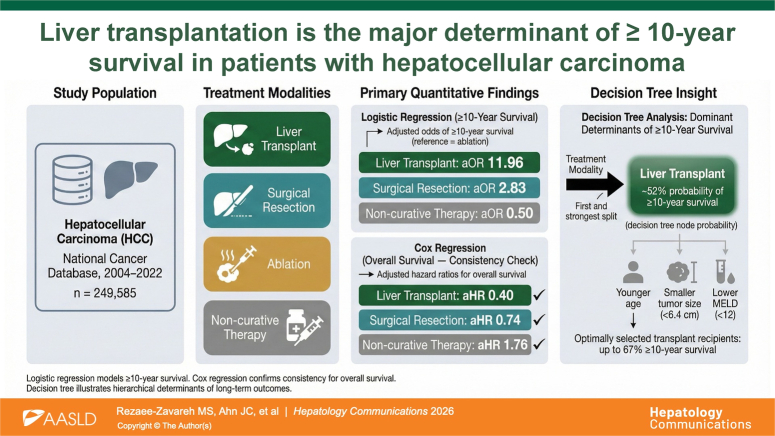

## INTRODUCTION

Hepatocellular carcinoma (HCC) is the most common primary liver cancer, primarily affecting patients with chronic liver diseases.^1^ HCC is among the leading causes of cancer incidence and mortality globally, and its incidence is projected to increase by 55% over the next 20 years.^2^ The prognosis depends heavily on the stage at diagnosis and treatments. Early diagnosis is critical, as curative therapies are primarily available for patients with early-stage disease.^3^^–^^5^

Curative HCC treatments include local ablation, surgical resection, or liver transplantation (LT).^3^^,^^4^ However, tumor recurrence is frequent, exceeding 50% at 5 years, in patients treated with ablation and resection due to pre-existing microscopic tumors from intrahepatic metastasis as well as the “field defect” in the background liver, which is prone to developing de novo HCC. Conversely, LT completely removes the diseased liver and replaces it with a healthy one, significantly reducing the cancer recurrence by ~10% in well-selected patients, as well as reducing the risk of mortality from hepatic decompensation. However, LT carries a higher morbidity and mortality risk in the first year compared with ablation and resection and requires lifelong immunosuppression. Further, there is a shortage of available donor organs compared with the number of patients on the waitlist.^6^

Several studies have compared the effectiveness of ablation, resection, and LT, with LT offering the highest overall survival (OS).^7^^–^^9^ However, most were limited to follow-up periods of up to 5 years, and none of them compared long-term survival beyond 10 years.

This study aims to identify factors associated with ≥10-year survival in HCC patients, focusing on treatment type. We also aim to evaluate the racial/ethnic disparity in long-term survival and treatment access.

## METHODS

### Database

We conducted a retrospective cohort study using the National Cancer Database (NCDB), which captures ~70% of newly diagnosed cancer cases in the United States.^10^ The NCDB, one of the largest cancer registries globally, has been jointly sponsored by the Commission on Cancer of the American College of Surgeons and the American Cancer Society since 1988. It encompasses national clinical oncology data from more than 1500 facilities approved by the Commission on Cancer.

### Patients and variables

The study included HCC patients diagnosed between 2004 and 2022. HCC-specific variables, including tumor size, stage, grade, and treatment, were extracted from NCDB. Curative treatment was defined as receipt of local ablation, surgical resection, or LT. NCDB treatment variables primarily capture first-course treatments; however, surgical therapies are recorded hierarchically, with LT, surgical resection, and ablation prioritized as more definitive treatments. As such, when surgical treatment is performed after an initial non-surgical therapy, the surgical procedure is still captured, whereas other subsequent therapies are not comprehensively recorded. In addition, we retrieved covariates on patient characteristics including age, sex, race and ethnicity, medical comorbidities, education level, income, treatment facility type, and location. Race and ethnicity were categorized as White, Hispanic, Black, Asian, and others. Medical comorbidities were graded by the Charlson/Deyo comorbidity index. Treatment facility type was classified into academic (>500 new cancer diagnoses annually and at least 4 postgraduate training programs) or non-academic (including comprehensive community, integrated networks, and community programs). Treating facilities were classified by geographic regions within the United States (Northeast, Midwest, South, and West). The study was based on de-identified data from the NCDB, so no direct patient contact, informed consent, or institutional review board approval was required.

### Statistical analysis

Baseline demographic and clinical characteristics were summarized as median with interquartile range (IQR) for continuous variables and as n (%) for categorical data. We obtained *p*-value*s* using the Kruskal–Wallis rank-sum test for continuous variables and the Pearson chi-squared test for categorical variables. Patients were stratified by length of survival into 4 groups: died ≤5 years, died 5–10 years, survived <10 years, and ≥10 years of follow-up. The primary outcome was survival ≥10 years. Independent variables included age, sex, race/ethnicity, tumor size, American Joint Committee on Cancer (AJCC) stage, comorbidities, socioeconomic status (income level), facility type, and treatment modality (LT, surgical resection, or ablation).

Multivariable logistic regression was employed to identify factors predicting ≥10-year survival, based on our predefined binary outcome (≥10 y vs. <5 y), adjusting for potential confounders. In this analysis, cases who died between 5 and 10 years and cases who were alive with <10 years of follow-up were excluded. This approach was chosen because the study objective focused specifically on long-term (≥10 y) survival rather than modeling time-to-event outcomes. To address potential concerns related to censoring, we also performed Cox proportional hazards analyses, including all death or alive cases with any follow-up time. Missing data were imputed using the multiple imputation with chained equations (MICE) algorithm.

A Sankey diagram was prepared, and a decision tree analysis was performed to identify the key determinants of long-term survival. A subgroup analysis was conducted among patients with early-stage HCC (AJCC T1), and those who received curative treatment. To ensure consistent results in patients with at least 10 years of follow-up, a sensitivity analysis was performed, excluding those diagnosed with HCC after 2011. In the decision tree and logistic regression analyses, cases who died between 5 and 10 years or were alive with <10 years of follow-up were excluded; all cases were included in the Sankey diagram and Cox analyses.

## RESULTS

### Patient characteristics

**FIGURE 1:**
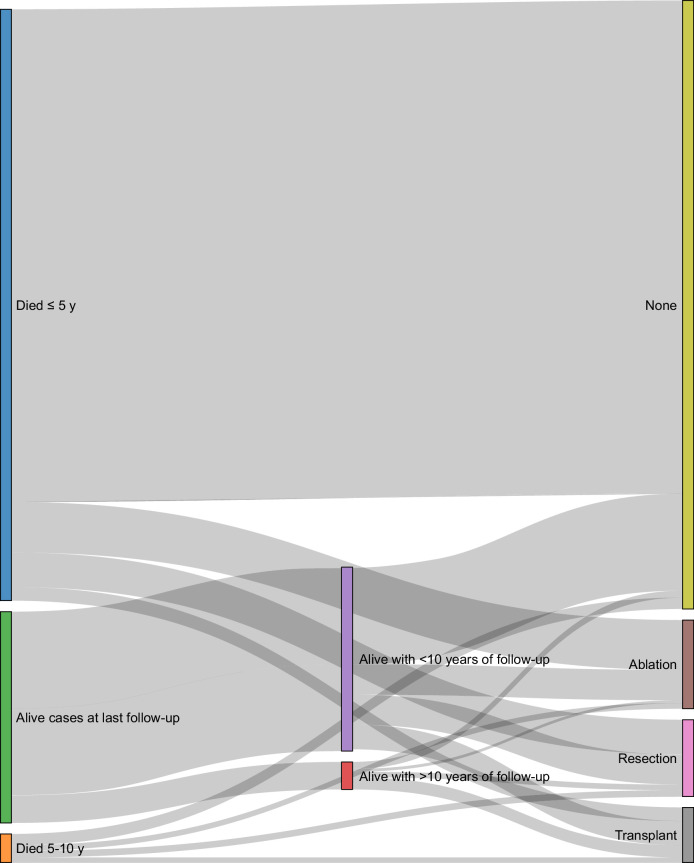
Distribution of survival outcomes by surgical treatment in patients with hepatocellular carcinoma. Sankey diagram showing survival outcomes by curative treatment modality. Flow widths are proportional to the number of patients. The green part shows alive cases, including those (n=54,988, 87%) with <10 years of follow-up (purple) and those (n=8219, 13%) with >10 years of follow-up (red). Blue and orange represent patients who died within 5 years and 5–10 years, respectively. In this diagram, all cases (alive or deceased, with any follow-up time) were included. Patients who died within 5 years predominantly received no curative treatment (83.4%), with smaller proportions undergoing ablation (8.4%), resection (5.9%), or transplant (2.3%). Among those who died between 5 and 10 years, no curative treatment remained the most common approach (39.6%), followed by resection (22.3%), ablation (19.4%), and transplant (18.7%). Patients who were alive with <10 years of follow-up received no curative treatment, ablation, resection, or transplantation in 53.3%, 16.7%, 16.6%, and 13.3% of cases, respectively. In contrast, among cases alive with more than 10 years of follow-up, the corresponding percentages were 25.6%, 10.5%, 20.1%, and 43.9%, respectively, highlighting liver transplantation as the most common approach in long-term survivors.

Table 1 shows the baseline characteristics of the NCDB cohort, stratified by survival categories: among 249,585 HCC patients from 2004 to 2022, 177,765 (71.2%) died within 5 years, 8613 (3.5%) died at 5–10 years, 54,988 (22.0%) were alive with <10 years of follow-up, and 8219 (3.3%) survived ≥10 years. Curative procedures were observed in 65,972 (27%) patients, including 26,517 (11%) who received local ablation, 22,967 (9.3%) surgical resection, and 16,488 (6.6%) LT. Among patients who survived ≥10 years, 44% received LT, 30% underwent resection or ablation, and 26% received no curative treatment. Also, based on the Sankey diagram (Figure 1), patients who received curative treatments (resection or LT) were more likely to have long-term survival. Among patients alive with more than 10 years of follow-up, LT remained the most common approach, whereas most patients who died within 5 years did not receive curative treatment.

**TABLE 1:** Baseline characteristics

Characteristic	Overall (N=249,585)	Died ≤5 y (N=177,765)	Died 5–10 y (N=8,613)	Alive <10 y F/U (N=54,988)	Alive ≥ 10 y F/U (N=8,219)	*p*
Age	64 (57, 72)	64 (57, 73)	63 (57, 71)	63 (58, 69)	57 (52, 62)	<0.001
Sex (male)	189,778 (76)	136,808 (77)	6432 (75)	40,578 (74)	5960 (73)	<0.001
Race/ethnicity						<0.001
White	151,467 (63)	110,164 (64)	5411 (65)	31,150 (58)	4742 (61)	
Hispanic	31,404 (13)	20,979 (12)	939 (11)	8418 (16)	1068 (14)	
Black	36,267 (15)	26,611 (16)	1159 (14)	7687 (14)	810 (10)	
Asian + Others	21,564 (9.0)	13,265 (7.8)	796 (9.6)	6290 (12)	1213 (15)	
Unknown	8883	6,746	308	1443	386	
Tumor size	44 (26, 75)	50 (30, 85)	31 (21, 50)	32 (21, 53)	28 (20, 43)	<0.001
Unknown	47,345	38,552	605	7738	450	
AJCC T staging						<0.001
T1	80,297 (40)	46,150 (33)	4290 (62)	26,234 (56)	3623 (59)	
T2	50,848 (25)	34,699 (25)	1895 (27)	12,249 (26)	2005 (33)	
T3	55,315 (28)	47,901 (34)	645 (9.4)	6298 (13)	471 (7.7)	
T4	13,543 (6.8)	11,538 (8.2)	66 (1.0)	1893 (4.1)	46 (0.7)	
Unknown	49,582	37,477	1717	8314	2074	
AJCC N staging						<0.001
N0	183,225 (91)	124,175 (88)	6809 (98)	46,150 (96)	6091 (98)	
N1	19,017 (9.4)	16,879 (12)	158 (2.3)	1859 (3.9)	121 (1.9)	
Unknown	47,343	36,711	1646	6979	2007	
AJCC M staging						<0.001
M0	196,155 (86)	133,752 (82)	7669 (98)	47,862 (95)	6872 (99)	
M1	31,791 (14)	28,843 (18)	127 (1.6)	2717 (5.4)	104 (1.5)	
Unknown	21,639	15,170	817	4409	1243	
Comorbidity score						<0.001
0	119,565 (48)	85,182 (48)	4075 (47)	26,446 (48)	3862 (47)	
1	57,460 (23)	40,564 (23)	2306 (27)	12,409 (23)	2181 (27)	
2	25,593 (10)	18,001 (10)	941 (11)	5955 (11)	696 (8.5)	
≥3	46,967 (19)	34,018 (19)	1291 (15)	10,178 (19)	1480 (18)	
Grade						<0.001
1	29,036 (33)	18,628 (31)	1647 (38)	7035 (36)	1726 (37)	
2	39,742 (45)	26,136 (43)	2081 (48)	9295 (48)	2230 (48)	
3	18,650 (21)	14,667 (24)	549 (13)	2828 (15)	606 (13)	
4	1102 (1.2)	900 (1.5)	30 (0.7)	124 (0.6)	48 (1.0)	
Unknown	161,055	117,434	4306	35,706	3609	
Insurance						<0.001
Medicaid/Medicare	154,976 (63)	114,384 (66)	5038 (60)	32,480 (60)	3074 (38)	
Private	72,029 (29)	46,432 (27)	2954 (35)	18,043 (33)	4600 (57)	
Not insured	12,722 (5.2)	9933 (5.7)	251 (3.0)	2317 (4.3)	221 (2.8)	
Other	5002 (2.0)	3432 (2.0)	175 (2.1)	1256 (2.3)	139 (1.7)	
Unknown	4856	3584	195	892	185	
Education (No HSD)						<0.001
15.3+	67,063 (31)	48,139 (31)	2080 (28)	14,814 (32)	2030 (28)	
9.1–15.2	64,067 (29)	46,768 (30)	2168 (29)	13,183 (28)	1948 (27)	
5.0–9.0	54,025 (25)	38,878 (25)	1987 (26)	11,297 (24)	1863 (26)	
<5.0	32,881 (15)	22,772 (15)	1303 (17)	7377 (16)	1429 (20)	
Unknown	31,549	21,208	1075	8317	949	
Income						<0.001
<$46,277	49,969 (23)	37,374 (24)	1510 (20)	9882 (21)	1203 (17)	
$46,277–$57,856	49,403 (23)	36,603 (23)	1629 (22)	9770 (21)	1401 (19)	
$57,857–$74,062	51,106 (24)	36,574 (23)	1810 (24)	11,062 (24)	1660 (23)	
$74,063+	66,941 (31)	45,548 (29)	2566 (34)	15,844 (34)	2983 (41)	
Unknown	32,166	21,666	1098	8430	972	
Facility type (non-academic)	118,622 (48)	91,153 (51)	3038 (35)	22,289 (41)	2142 (26)	<0.001
Location						<0.001
Northeast	50,256 (20)	34,654 (20)	1825 (21)	11,826 (22)	1951 (25)	
Midwest	50,207 (20)	37,029 (21)	1877 (22)	9746 (18)	1555 (20)	
South	96,820 (39)	69,734 (40)	3077 (36)	21,301 (40)	2708 (34)	
West	48,862 (20)	34,519 (20)	1735 (20)	10,883 (20)	1725 (22)	
Unknown	3440	1829	99	1232	280	
MELD	12 (8, 20)	13 (9, 21)	10 (7, 16)	10 (7, 17)	11 (8, 16)	<0.001
Unknown	171,885	122,750	5229	37,836	6070	
Cirrhosis						<0.001
No	9375 (23)	5,801 (21)	676 (29)	2254 (25)	644 (27)	
Unknown	208,912	150,721	6294	46,038	5859	
AFP						<0.001
Negative	39,532 (27)	25,109 (23)	2781 (43)	9415 (39)	2227 (37)	
Unknown	102,095	66,984	2121	30,818	2172	
Curative treatment						<0.001
Ablation	26,517 (11)	14,880 (8.4)	1660 (19)	9121 (17)	856 (10)	
Resection	22,967 (9.3)	10,366 (5.9)	1906 (22)	9057 (17)	1638 (20)	
Transplant	16,488 (6.6)	4026 (2.3)	1605 (19)	7271 (13)	3586 (44)	
Non-curative treatment	182,027 (73)	147,522 (83)	3393 (40)	29,023 (53)	2089 (26)	
Unknown	1586	971	49	516	50	

*Note:* Data are presented as median (IQR) or n (%). Statistical tests were based on the Kruskal–Wallis rank-sum test and the Pearson chi-squared test. Groups compared included patients who died within 5 years of follow-up, those who died between 5 and 10 years, those alive with <10 years of follow-up, and those alive with 10 or more years of follow-up.

Abbreviations: AFP, alpha-fetoprotein; AJCC, American Joint Committee on Cancer; F/U, follow-up; HSD, high school diploma; MELD, Model for End-stage Liver Disease.

Compared with the other survival groups, patients who survived ≥10 years had a significantly lower median age (57 y; IQR, 52–62) and smaller median tumor size (28 mm; IQR, 20–43) (*p* < 0.001 for both). Black individuals (10%), those with more advanced stages (T3: 7.7% and T4: 0.7%), and those with higher Charlson/Deyo comorbidity scores (2: 8.5% and ≥3: 18%) comprised the lowest proportions in this group compared with the other survival groups (*p* < 0.001 for all). Those who survived ≥10 years also had the highest proportion of cases with private insurance (57%) compared with the other survival groups (≤35%; *p* < 0.001).

### Factors associated with over 10 years of survival in multivariable logistic regression

The logistic regression analysis unveiled a complex interplay of factors influencing long-term survival in HCC patients (Table 2). Treatment modality emerged as the most potent predictor; compared with local ablation, both LT [adjusted odds ratio (aOR): 11.96, 95% confidence interval (CI): 11.27–13.29, *p*<0.001] and surgical resection (aOR: 2.83, 95% CI: 2.57–3.08, *p*<0.001) were associated with improved odds of ≥10-year survival. Compared with resection, LT was associated with significantly improved odds of ≥10-year survival (aOR 4.23, 95% CI: 3.74–4.78). Patients receiving non-curative treatment faced substantially reduced odds of long-term survival. Demographic factors were also associated with long-term survival, with younger age, female sex, and Asian or Hispanic race/ethnicity associated with improved survival, while Black individuals were associated with lower odds of ≥10-year survival (aOR: 0.88, 95% CI: 0.82–0.96) compared with Whites. Tumor characteristics, particularly smaller size and earlier AJCC stage, were linked to better survival. Socioeconomic factors also proved influential, as patients with private insurance and those from higher-income areas demonstrated increased odds of long-term survival. The type of treatment facility mattered, with academic centers associated with better outcomes. In addition, geographic disparities were observed, with patients in the Northeast faring better than those in other regions. Clinical factors such as lower MELD scores, negative AFP, and no cirrhosis status were all associated with improved long-term survival.

**TABLE 2:** Logistic regression model for determining factors affecting ≥10-year survival among patients with hepatocellular carcinoma

Characteristic	Univariate		Multivariable	
	OR (95% CI)	*p*	OR (95% CI)	*p*
Age (10 y)	0.54 (0.53, 0.55)	<0.001	0.58 (0.56, 0.59)	<0.001
Sex				
Male	—		—	
Female	1.23 (1.18, 1.29)	<0.001	1.36 (1.28, 1.43)	<0.001
Race/ethnicity				
White	—		—	
Hispanic	1.11 (1.04, 1.18)	0.001	1.26 (1.19, 1.39)	<0.001
Black	0.70 (0.66, 0.75)	<0.001	0.88 (0.82, 0.96)	0.002
Asian + Others	1.99 (1.87, 2.11)	<0.001	1.91 (1.76, 2.06)	<0.001
Tumor size (cm)	0.80 (0.79, 0.81)	<0.001	0.93 (0.92, 0.94)	<0.001
AJCC T staging				
T1	—		—	
T2	0.72 (0.66, 0.73)	<0.001	0.71 (0.64, 0.72)	<0.001
T3	0.11 (0.10, 0.12)	<0.001	0.32 (0.28, 0.34)	<0.001
T4	0.04 (0.03, 0.05)	<0.001	0.16 (0.11, 0.18)	<0.001
AJCC N staging				
N0	—		—	
N1	0.13 (0.12, 0.15)	<0.001	0.54 (0.49, 0.67)	<0.001
AJCC M staging				
M0	—		—	
M1	0.07 (0.06, 0.08)	<0.001	0.32 (0.27, 0.38)	<0.001
Comorbidity score				
0	—		—	
1	1.21 (1.15, 1.27)	<0.001	1.09 (1.03, 1.17)	0.004
2	0.88 (0.81, 0.94)	<0.001	0.86 (0.79, 0.94)	<0.001
≥3	0.95 (0.90, 1.01)	0.090	0.75 (0.71, 0.82)	<0.001
Insurance				
Medicaid/Medicare	—		—	
Private	3.35 (3.22, 3.50)	<0.001	1.74 (1.66, 1.85)	<0.001
Not insured	0.73 (0.64, 0.82)	<0.001	0.90 (0.77, 1.02)	0.160
Other	1.47 (1.26, 1.71)	<0.001	1.28 (1.06, 1.53)	0.008
Education (% No HSD)				
15.3%+	—		—	
9.1%–15.2%	0.99 (0.93, 1.04)	0.700	0.88 (0.82, 0.95)	<0.001
5.0%–9.0%	1.17 (1.11, 1.24)	<0.001	0.91 (0.85, 1.00)	0.018
<5.0%	1.48 (1.41, 1.59)	<0.001	0.98 (0.91, 1.10)	0.731
Income				
<$46,277	—		—	
$46,277–$57,856	1.21 (1.13, 1.29)	<0.001	1.16 (1.06, 1.25)	<0.001
$57,857–$74,062	1.41 (1.31, 1.49)	<0.001	1.26 (1.15, 1.36)	<0.001
$74,063+	1.97 (1.86, 2.09)	<0.001	1.57 (1.40, 1.67)	<0.001
Facility type				
Non-academic	—		—	
Academic	2.94 (2.81, 3.08)	<0.001	1.63 (1.54, 1.72)	<0.001
Location				
Northeast	—		—	
Midwest	0.81 (0.77, 0.87)	<0.001	0.77 (0.72, 0.84)	<0.001
South	0.74 (0.70, 0.78)	<0.001	0.77 (0.73, 0.84)	<0.001
West	0.94 (0.88, 1.00)	0.044	0.89 (0.83, 0.96)	0.003
MELD (10 units)	0.82 (0.82, 0.82)	<0.001	0.85 (0.84, 0.85)	<0.001
Cirrhosis status				
Non-cirrhotic	—		—	
Cirrhotic	0.72 (0.70, 0.77)	<0.001	0.64 (0.58, 0.65)	<0.001
AFP				
Negative	—		—	
Positive	0.50 (0.47, 0.51)	<0.001	0.78 (0.72, 0.80)	<0.001
Treatment				
Ablation	—		—	
Resection	2.70 (2.50, 2.93)	<0.001	2.83 (2.57, 3.08)	<0.001
Transplant	15.20 (14.10, 16.41)	<0.001	11.96 (11.27, 13.29)	<0.001
Non-curative treatment	0.23 (0.22, 0.25)	<0.001	0.50 (0.47, 0.55)	<0.001

*Note:* Univariable and multivariable logistic regression analyses were performed to assess survival as a binary outcome (≥10 y vs. <5 y), and data are presented as odds ratios (ORs) with 95% confidence intervals (CIs). In this analysis, cases who died between 5 and 10 years and cases who were alive with <10 years of follow-up were excluded.

Abbreviations: AFP, alpha-fetoprotein; AJCC, American Joint Committee on Cancer; CI, confidence interval; HSD, high school diploma; MELD, Model for End-stage Liver Disease; OR, odds ratio.

The logistic regression analysis excluded cases who died between 5 and 10 years or were alive with <10 years of follow-up. To account for survival time more comprehensively, a Cox proportional hazards regression analysis including all cases, regardless of vital status or follow-up time, was also performed (Supplemental Table S1, http://links.lww.com/HC9/C337). The results showed that, compared with local ablation, both LT [adjusted hazard ratio (aHR): 0.40, 95% CI: 0.39–0.41, *p*<0.001] and surgical resection (aHR: 0.74, 95% CI: 0.72–0.76, *p*<0.001) were associated with improved OS, whereas non-curative treatment was associated with lower OS (aHR: 1.76, 95% CI: 1.73–1.79, *p*<0.001).

### Factors associated with over 10 years of survival in T1 HCC patients

In the subgroup analysis of patients with T1 tumors, similar trends were observed (Table 3). Notably, the impact of treatment modality was even more pronounced in this subgroup. Compared with ablation, LT was associated with 11.47 times higher odds of ≥10-year survival (95% CI: 10.35–13.09, *p*<0.001), while surgical resection was associated with 3.16 times higher odds (95% CI: 2.72–3.50, *p*<0.001). Compared with surgical resection, LT was associated with 3.63 times higher odds of ≥10-year survival (95% CI: 3.02–4.36, *p*<0.001). Racial and ethnic disparities persist in T1 HCC subgroup analysis: Asian or Hispanic individuals were associated with improved survival, while Black individuals had lower odds of ≥10-year survival (aOR: 0.88, 95% CI: 0.77–0.99). In a sensitivity analysis excluding patients diagnosed after 2011, the results remained consistent with LT (aOR 8.43, 95% CI: 7.62–9.19, *p*<0.001) and surgical resection (aOR 2.39, 95% CI: 2.21–2.70, *p*<0.001) having significantly higher odds of ≥10-year survival compared with ablation (Supplemental Table S2, http://links.lww.com/HC9/C337).

**TABLE 3:** Logistic regression model for determining factors affecting ≥10-year survival among patients with hepatocellular carcinoma categorized as T1 based on the American Joint Committee on Cancer staging

Characteristic	Univariate		Multivariable	
	OR (95% CI)	*p*	OR (95% CI)	*p*
Age (10 y)	0.49 (0.47, 0.51)	<0.001	0.55 (0.53, 0.57)	<0.001
Sex				
Male	—		—	
Female	1.04 (0.97, 1.11)	0.308	1.30 (1.20, 1.42)	<0.001
Race/ethnicity				
White	—		—	
Hispanic	1.12 (1.02, 1.24)	0.023	1.27 (1.15, 1.46)	<0.001
Black	0.85 (0.76, 0.94)	0.002	0.88 (0.77, 0.99)	0.041
Asian + Others	2.44 (2.22, 2.68)	<0.001	2.15 (1.89, 2.40)	<0.001
Tumor size (cm)	0.85 (0.84, 0.87)	<0.001	0.90 (0.89, 0.91)	<0.001
Comorbidity score				
0	—		—	
1	1.19 (1.10, 1.28)	<0.001	1.08 (0.99, 1.19)	0.099
2	0.88 (0.79, 0.98)	0.020	0.88 (0.78, 1.02)	0.047
≥3	0.90 (0.83, 0.99)	0.025	0.72 (0.65, 0.81)	<0.001
Insurance				
Medicaid/Medicare	—		—	
Private	3.66 (3.46, 3.94)	<0.001	1.81 (1.69, 1.98)	<0.001
Not Insured	0.85 (0.68, 1.05)	0.152	0.75 (0.59, 0.95)	0.019
Other	1.38 (1.07, 1.72)	0.007	1.29 (0.97, 1.66)	0.061
Education (% No HSD)				
15.3%+	—		—	
9.1%–15.2%	1.04 (0.94, 1.12)	0.410	0.95 (0.87, 1.07)	0.302
5.0%–9.0%	1.18 (1.09, 1.29)	<0.001	0.95 (0.87, 1.10)	0.384
<5.0%	1.46 (1.31, 1.58)	<0.001	1.01 (0.89, 1.19)	0.910
Income				
<$46,277	—		—	
$46,277–$57,856	1.15 (1.01, 1.25)	0.008	1.10 (0.95, 1.21)	0.124
$57,857–$74,062	1.40 (1.23, 1.50)	<0.001	1.21 (1.03, 1.32)	0.004
$74,063+	2.04 (1.83, 2.19)	<0.001	1.57 (1.31, 1.72)	<0.001
Facility type				
Non-academic	—		—	
Academic	2.38 (2.22, 2.55)	<0.001	1.56 (1.44, 1.70)	<0.001
Location				
Northeast	—		—	
Midwest	0.73 (0.67, 0.81)	<0.001	0.73 (0.65, 0.82)	<0.001
South	0.66 (0.60, 0.72)	<0.001	0.70 (0.64, 0.79)	<0.001
West	0.89 (0.82, 0.98)	0.013	0.82 (0.75, 0.93)	<0.001
MELD (10 units)	0.85 (0.79, 0.85)	<0.001	0.86 (0.79, 0.86)	<0.001
Cirrhosis status				
Non-cirrhotic	—		—	
Cirrhotic	0.70 (0.64, 0.74)	<0.001	0.63 (0.54, 0.65)	<0.001
AFP				
Negative	—		—	
Positive	0.74 (0.70, 0.79)	<0.001	0.81 (0.75, 0.88)	<0.001
Curative treatment				
Ablation	—		—	
Resection	3.03 (2.72, 3.38)	<0.001	3.16 (2.72, 3.50)	<0.001
Transplant	14.37 (12.91, 16.04)	<0.001	11.47 (10.35, 13.09)	<0.001
Non-curative treatment	0.41 (0.37, 0.45)	<0.001	0.55 (0.50, 0.61)	<0.001

*Note:* Univariable and multivariable logistic regression analyses were performed to assess survival as a binary outcome (≥10 y vs. <5 y), and data are presented as odds ratios (ORs) with 95% confidence intervals (CIs). In this analysis, cases who died between 5 and 10 years and cases who were alive with <10 years of follow-up were excluded.

Abbreviations: AFP, alpha-fetoprotein; AJCC, American Joint Committee on Cancer; CI, confidence interval; HSD, high school diploma; MELD, Model for End-stage Liver Disease; OR, odds ratio.

### Major determinant for ≥10-year survival in decision tree analysis

**FIGURE 2:**
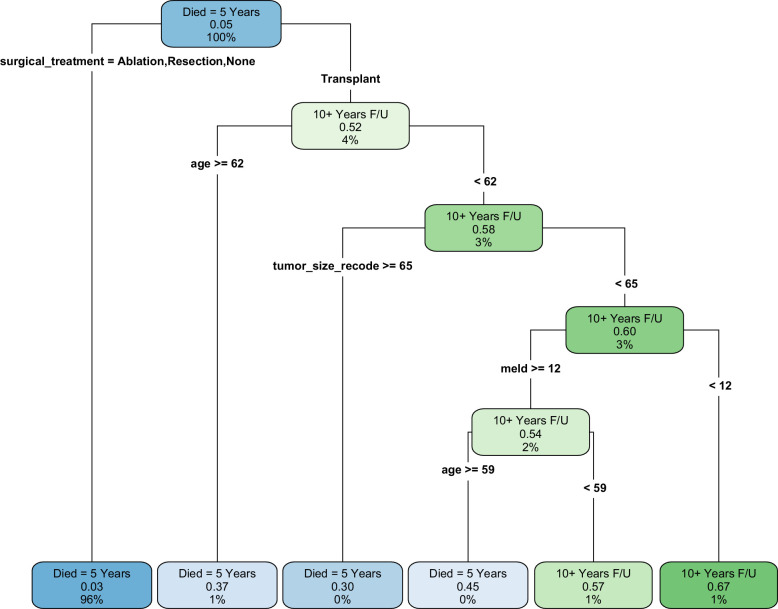
Major determinants of ≥10-year survival in patients with hepatocellular carcinoma based on decision tree analysis in the entire cohort. Decision tree showing major determinants of ≥10-year survival in all patients with hepatocellular carcinoma. In this analysis, cases who died between 5 and 10 years and cases who were alive with <10 years of follow-up were excluded. Each node displays: the dominant outcome (top), the probability of ≥10-year survival (middle; eg, 0.52=52%), and the percentage of the total cohort represented by that node (bottom). Green nodes indicate subgroups in which ≥10-year survival is the more likely outcome (survival probability >50%), while blue nodes indicate subgroups in which death within 5 years is more likely (survival probability <50%). LT emerged as the most favorable treatment option, with a 52% probability of ≥10-year survival (4% of the total cohort). Within the LT pathway, younger age, smaller tumor size, and lower MELD score were associated with progressively higher probabilities of ≥10-year survival, reaching 67% in patients <62 years with tumor size <6.4 cm and MELD score <12 (1% of the cohort). In contrast, most non-transplant treatment pathways remained dominated by early mortality (blue nodes). A split variable (age, tumor size, MELD, etc.) partitions a node into 2 subgroups. If both resulting subgroups still have a majority probability for the same outcome (both have ≥50% chance of long-term survival, or both have <50%), then both child boxes will keep the same color as the parent (they differ only in the actual probability value). Example: A blue parent (majority die ≤5 y) may split into 2 blue children if neither subgroup reaches ≥50% long-term survival. If one child subgroup crosses the 50% threshold in the opposite direction, then one child becomes green and the other blue. Abbreviations: F/U, follow-up; LT, liver transplantation; MELD, Model for End-stage Liver Disease.

To further elucidate the factors most critical in determining long-term survival, we performed decision tree analyses for the entire cohort, the subset of patients with T1 tumors, and also for cases who received curative treatment. The decision tree for all patients revealed that the type of treatment was the primary determinant of survival outcomes (Figure 2). LT emerged as the most favorable treatment option, associated with a 52% probability of ≥10-year survival. Other factors associated with ≥10-year survival included younger age, smaller tumor size, and lower MELD score.

For patients with T1 tumors (Supplemental Figure S1A, http://links.lww.com/HC9/C337) and for those who underwent curative treatment (Supplemental Figure S1B, http://links.lww.com/HC9/C337), separate decision tree analyses were performed to identify major determinants of ≥10-year survival, highlighting LT as the primary predictor of long-term survival. Overall, these decision tree analyses reinforced the findings from the logistic and Cox regression, emphasizing the critical role of LT in achieving long-term survival for patients with HCC, particularly those with early-stage (T1) tumors.

### Factors associated with liver transplant receipt

Multiple common factors were associated with a higher likelihood of LT receipt (Table 4). These include younger age, higher Charlson/Deyo comorbidity score, earlier stage HCC with lower tumor burden, having private insurance, and receiving care at an academic center. Asian and others, as well as Hispanic individuals, had similar access to LT, whereas Black individuals had a lower likelihood of receiving a transplant compared with Whites (aOR: 0.73, 95% CI: 0.55–0.96, *p*=0.026).

**TABLE 4:** Logistic regression model for determining factors associated with liver transplant receipt among patients with hepatocellular carcinoma

Characteristic	Univariate		Multivariable	
	OR (95% CI)	*p*	OR (95% CI)	*p*
Age (10 y)	0.56 (0.55, 0.58)	<0.001	0.73 (0.65, 0.81)	<0.001
Sex				
Male	—		—	
Female	0.91 (0.87, 0.96)	<0.001	0.85 (0.70, 1.04)	0.11
Race/ethnicity				
White	—		—	
Hispanic	0.88 (0.83, 0.94)	<0.001	1.00 (0.77, 1.29)	>0.9
Black	0.56 (0.52, 0.60)	<0.001	0.73 (0.55, 0.96)	0.026
Asian + Others	0.88 (0.81, 0.95)	0.001	1.18 (0.87, 1.57)	0.3
Tumor size (cm)	0.70 (0.70, 0.71)	<0.001	0.82 (0.78, 0.87)	<0.001
AJCC T staging				
T1	—		—	
T2	1.04 (0.99, 1.09)	0.15	0.97 (0.82, 1.14)	0.7
T3	0.10 (0.09, 0.11)	<0.001	0.14 (0.08, 0.22)	<0.001
T4	0.05 (0.03, 0.06)	<0.001	0.15 (0.01, 0.71)	0.063
AJCC N staging				
N0	—		—	
N1	0.09 (0.07, 0.11)	<0.001	0.29 (0.09, 0.71)	0.018
AJCC M staging				
M0	—		—	
M1	0.03 (0.02, 0.04)	<0.001	0.17 (0.04, 0.46)	0.003
Comorbidity score				
0	—		—	
1	1.46 (1.38, 1.54)	<0.001	1.75 (1.41, 2.19)	<0.001
2	1.27 (1.18, 1.36)	<0.001	1.59 (1.19, 2.10)	0.001
≥3	1.91 (1.82, 2.01)	<0.001	2.10 (1.70, 2.60)	<0.001
Insurance				
Medicaid/Medicare	—		—	
Private	2.96 (2.84, 3.09)	<0.001	2.49 (2.09, 2.96)	<0.001
Not insured	0.40 (0.33, 0.47)	<0.001	0.48 (0.25, 0.83)	0.014
Other	1.29 (1.10, 1.51)	0.002	0.52 (0.22, 1.04)	0.094
Education (% No HSD)				
15.3%+	—		—	
9.1%–15.2%	1.20 (1.13, 1.27)	<0.001	1.06 (0.84, 1.34)	0.6
5.0%–9.0%	1.33 (1.25, 1.41)	<0.001	1.06 (0.81, 1.38)	0.7
<5.0%	1.55 (1.45, 1.66)	<0.001	1.12 (0.82, 1.53)	0.5
Income				
<$46,277	—		—	
$46,277–$57,856	1.26 (1.18, 1.35)	<0.001	0.94 (0.72, 1.23)	0.7
$57,857–$74,062	1.34 (1.25, 1.43)	<0.001	0.87 (0.65, 1.15)	0.3
$74,063+	1.61 (1.51, 1.72)	<0.001	1.08 (0.80, 1.45)	0.6
Facility type				
Non-academic	—		—	
Academic	4.15 (3.95, 4.37)	<0.001	1.99 (1.63, 2.44)	<0.001
Location				
Northeast	—		—	
Midwest	1.29 (1.21, 1.38)	<0.001	1.67 (1.26, 2.22)	<0.001
South	1.13 (1.07, 1.20)	<0.001	1.84 (1.44, 2.38)	<0.001
West	0.93 (0.87, 1.00)	0.043	0.91 (0.70, 1.17)	0.4
MELD (10 units)	1.11 (1.07, 1.14)	<0.001	1.23 (1.14, 1.33)	<0.001
Cirrhosis status				
Non-cirrhotic	—		—	
Cirrhotic	1.44 (1.30, 1.58)	<0.001	1.10 (0.86, 1.42)	0.5
AFP				
Negative	—		—	
Positive	0.51 (0.48, 0.54)	<0.001	0.90 (0.76, 1.07)	0.2

*Note:* Univariable and multivariable logistic regression analyses were performed to assess survival as a binary outcome (≥10 y vs. <5 y), and data are presented as odds ratios (ORs) with 95% confidence intervals (CIs). In this analysis, patients who died between 5 and 10 years and patients who were alive with <10 years of follow-up were excluded.

Abbreviations: AFP, alpha-fetoprotein; AJCC, American Joint Committee on Cancer; CI, confidence interval; HSD, high school diploma; MELD, Model for End-stage Liver Disease; OR, odds ratio.

## DISCUSSION

This large-scale retrospective cohort study of the NCDB provides compelling evidence that surgical therapy, particularly LT, is the major determinant of achieving long-term survival for patients with HCC. The analysis demonstrated that even after adjusting for various demographic, clinical, and socioeconomic factors, LT was associated with nearly 12 times and 4 times higher odds of 10-year survival compared with local ablation and surgical resection, respectively. Importantly, this survival benefit was even more pronounced in patients with T1 tumors. The study also identified other factors significantly associated with long-term survival, including younger age, female sex, Asian or Hispanic race/ethnicity, smaller tumor size, and treatment at academic centers, providing a comprehensive picture of prognostic indicators in HCC management. Unfortunately, Black individuals had lower odds of ≥10-year survival and a decreased likelihood of receiving LT. Thus, ensuring equitable access to transplantation is crucial for this population.

In our study, the markedly higher odds of ≥10-year survival observed with LT (aOR: 11.47) in early-stage HCC aligns with previous studies showing it offers the best long-term outcomes, with 5-year and 10-year survival of 71.3% and 59%, respectively.^11^ The superiority of LT stems from several factors. Transplantation not only addresses possible microscopic intrahepatic metastases but also addresses the background field defect, thereby reducing the risk of de novo tumor formation,^12^^,^^13^ and yielding the lowest recurrence rates among the three treatment modalities. Studies show the recurrence rate after LT is ~10%–15% for patients meeting the Milan criteria, compared with much higher rates for resection and ablation.^14^ Further, LT reduces the risk of liver-related mortality. Although the short-term prognosis for Child–Pugh A cirrhosis patients is favorable, the risk of hepatic decompensation is ~20% over 2 years, with a 10% risk of death in the same period.

Superior long-term outcomes of LT over resection and ablation also highlight the value of downstaging HCC beyond standard criteria to enable transplantation. Ten-year post-LT survival after successful downstaging to within Milan criteria has been reported to be favorable.^11^ Even post-LT survival is comparable between patients meeting United Network for Organ Sharing (UNOS) downstaging criteria and those beyond it (All-Comers).^15^ Currently, locoregional therapies are recommended for this purpose,^16^ and systemic therapies like immune checkpoint inhibitors may increase the chance of patients receiving LT as a curative approach.^17^^–^^19^ However, this approach, which is also being evaluated after LT,^20^ may increase the risk of allograft rejection, and thus clinical trials are still needed.^21^

It is important to acknowledge the practical limitations of recommending LT for all HCC patients. Many face medical, social, or financial barriers to accessing curative therapies, including transplantation, surgical resection, and advanced ablative techniques. These barriers disproportionately affect racial/ethnic minorities and low socioeconomic populations, who are also more likely to be impacted by HCC.^22^^–^^24^ In these vulnerable populations, patients are more likely to present late in the disease process, often excluding LT as a curative option. Further, the scarcity of donor organs remains a significant barrier to widespread application of LT even for early-stage HCC which may be amenable to other treatments.^25^ This shortage necessitates careful patient selection and prioritization to ensure optimal utilization of this limited resource. The safety profiles and complication rates of each treatment modality should also be considered when interpreting our results. While LT offers the best long-term outcomes, it carries significant perioperative risks and long-term complications related to immunosuppression.^26^

Surgical resection and ablation, while associated with lower long-term survival compared with transplantation, still offer substantial benefits for selected patients. Our results show that resection is associated with higher odds of ≥10-year survival compared with ablation, suggesting it should be the preferred option when transplantation is not feasible and the patient has adequate liver function. This finding aligns with previous studies reporting 5-year survival rates of ~70%–80% for hepatic resection in early-stage HCC.^27^ However, recurrence rates after resection are significant, with studies reporting 1-year, 3-year, and 5-year rates of 19%, 54%, and 70%, respectively.^28^ The choice between resection and ablation often depends on tumor size, liver function, and patient comorbidities. For very early-stage HCC (single tumor ≤2 cm), radiofrequency ablation (RFA) is recommended as first-line treatment by international guidelines.^16^^,^^29^^–^^31^ For tumors 2–3 cm, both resection and RFA are viable options, with the choice often depending on the patient’s liver function and overall health. However, RFA is associated with higher local recurrence rates than surgical resection. When RFA is used as first-line therapy for early-stage HCC, studies report a 5-year recurrence rate of 50%–70%.^32^^,^^33^ Several ongoing studies explore (neo)adjuvant strategies. Effective (neo)adjuvant therapy may improve long-term survival after resection and ablation.

A significant disparity in LT access and outcomes persists among different racial/ethnic groups.^34^^,^^35^ Our study showed that Black individuals were associated with significantly reduced 10-year survival compared with other racial/ethnic groups. Furthermore, Black patients had a lower likelihood of receiving LT even after adjusting for demographic, socioeconomic, and clinical confounders. A recent study showed that Black HCC patients are more likely to be removed from the transplant list and experience the worst post-transplant survival.^35^ Another nationwide study in the United States, published in 2023, using the National Inpatient Sample (NIS) database, evaluated 112,110 adults with HCC, of whom 3020 underwent LT from 2016 to 2020. Black patients had a 40% (95% CI: 54–22) lower chance of receiving LT compared with White patients, and also experienced higher mortality and complications, including sepsis and acute kidney injury.^36^ Also, a 2025 analysis of 9677 LT admissions from this database (2016–2021) found that the majority of recipients were White (66–68%), followed by Hispanic (14–17%) and Black patients (7–10%). However, transplant rates relative to liver failure admissions remained consistent across racial groups. Also, the study found that in-hospital mortality ranged from 2.37% to 3.52% and did not significantly differ by race. It concluded that, while post-transplant outcomes were comparable across demographic and socioeconomic groups, access to transplantation continues to show racial disparities.^37^ Disparities in access to transplantation and long-term survival among Black individuals may stem from unmeasured/residual social determinants of health, as well as cultural and linguistic barriers that limit access to transplantation and other standards of care for HCC and underlying liver diseases.^38^ Other contributing factors may include alcohol or drug use, which disproportionately affected Black individuals, medical comorbidities and older age, and lack of referral for LT despite meeting criteria. In addition, some patients declined LT after referral.^39^^,^^40^ Together, these factors help explain the persistent racial disparities in LT for HCC. The impact of other socioeconomic factors on long-term survival is also noteworthy. For example, patients with private insurance and those from higher-income areas had significantly better odds of ≥10-year survival. This highlights the urgent need for policies and interventions to address disparities in access to optimal HCC care.

Our study has several strengths, including its large sample size, long follow-up, and use of a nationally representative database. However, it also has limitations inherent to retrospective studies using administrative databases. The NCDB lacks detailed information on liver function, portal hypertension, and specific tumor characteristics that may influence treatment selection and outcomes. Importantly, our study is subject to channeling bias, where treatment selection is influenced by factors not fully captured in the database. This bias could affect outcome comparison across treatment modalities, as patients undergoing transplantation, resection, ablation, or non-curative treatment may differ systematically in characteristics that impact long-term survival. Patients selected for transplantation are likely to be healthier in ways not fully captured by comorbidity indices or Eastern Cooperative Oncology Group-performance status, while those selected for resection may differ in nuanced aspects of liver function. For example, CTP class A patients with low MELD scores are not homogeneous. These unmeasured differences could systematically affect survival outcomes, especially when survival, rather than HCC recurrence, is the primary endpoint. Future studies should also determine if the survival benefit of transplantation over resection and ablation varies by etiology. For example, patients with metabolic dysfunction–associated steatotic liver disease (MASLD) who remain at continued risk of liver disease progression may benefit more from transplantation than those with hepatitis C virus infection who have achieved sustained virologic response and faced reduced risk of future hepatic decompensation. These limitations underscore the need for prospective studies to validate our findings and address confounding factors.

Our results have important implications for clinical practice and policy. They underscore the need for early referral of HCC patients to transplant centers and highlight the potential long-term benefits of transplantation in carefully selected patients. They also emphasize the continued importance of surgical resection and ablation as effective treatment options, where transplantation is not feasible.

Future research should focus on refining selection criteria for treatment modalities, developing strategies to expand the donor pool, and investigating novel approaches to bridge patients to transplantation while maintaining excellent long-term outcomes. In addition, prospective studies incorporating detailed clinical and biological data are needed to clarify factors contributing to very long-term survival in HCC patients.

## CONCLUSION

In conclusion, while LT is the optimal treatment for achieving long-term survival in HCC, a multidisciplinary approach considering patient factors, tumor characteristics, and available resources remains crucial for managing this complex disease. Substantial racial, ethnic, and socioeconomic disparities persist in access to optimal care and long-term outcomes for HCC patients. Urgent efforts are needed to develop strategies to reduce these disparities.

## Supplementary Material
