# Supplementary material for: Liver transplantation is the major determinant of ≥10-year survival in patients with hepatocellular carcinoma
Source: Hepatol Commun. 2026 Apr 24;10(5):e0951. doi: 10.1097/HC9.0000000000000951 (PMC13120506; doi:10.1097/HC9.0000000000000951)
Supplement: Supplementary file 1 [file hc9-10-e0951-s001.docx]

**Supplementary Table 1. Cox Proportional Hazards Regression Model for Determining Factors Associated with Overall Survival Among Patients with Hepatocellular Carcinoma**

| Characteristic | Univariate | | | Multivariable | | |
| --- | --- | --- | --- | --- | --- | --- |
|  | **HR***^1^* | **95% CI** | **p-value** | **HR** | **95% CI** | **p-value** |
| Age (10 Years) | 1.16 | 1.15,1.16 | <0.001 | 1.11 | 1.11, 1.12 | <0.001 |
| Sex (ref: male) |  |  |  |  |  |  |
| Female | 0.89 | 0.88, .90 | <0.001 | 0.95 | 0.94, 0.96 | <0.001 |
| Race / Ethnicity (ref: White) |  |  |  |  |  |  |
| Hispanic | 0.89 | 0.87, 0.90 | <0.001 | 0.83 | 0.82, 0.85 | <0.001 |
| Black | 1.06 | 1.04, 1.07 | <0.001 | 0.93 | 0.91, 0.94 | <0.001 |
| Asian + Others | 0.76 | 0.75, 0.77 | <0.001 | 0.82 | 0.80, 0.83 | <0.001 |
| Tumor Size (cm) | 1.03 | 1.03, 1.03 | <0.001 | 1.02 | 1.02, 1.02 | <0.001 |
| AJCC T Staging, (ref: T1) |  |  |  |  |  |  |
| T2 | 1.32 | 1.30, 1.34 | <0.001 | 1.24 | 1.22, 1.25 | <0.001 |
| T3 | 2.90 | 2.86, 2.93 | <0.001 | 1.80 | 1.76, 1.83 | <0.001 |
| T4 | 3.51 | 3.42, 3.61 | <0.001 | 1.93 | 1.87, 1.99 | <0.001 |
| AJCC N Staging (ref: N0) |  |  |  |  |  |  |
| N1 | 2.48 | 2.44, 2.52 | <0.001 | 1.25 | 1.23, 1.28 | <0.001 |
| AJCC M Staging (ref: M0) |  |  |  |  |  |  |
| M1 | 3.10 | 3.06, 3.14 | <0.001 | 1.70 | 1.67, 1.73 | <0.001 |
| Comorbidity Score (ref:0) |  |  |  |  |  |  |
| 1 | 0.96 | 0.95, 0.97 | <0.001 | 1.04 | 1.03, 1.05 | <0.001 |
| 2 | 0.97 | 0.96, 0.99 | <0.001 | 1.07 | 1.05, 1.09 | <0.001 |
| ≥3 | 1.09 | 1.08, 1.10 | <0.001 | 1.27 | 1.25, 1.28 | <0.001 |
| Insurance (ref: Medicaid/Medicare) |  |  |  |  |  |  |
| Private | 0.74 | 0.73, 0.74 | <0.001 | 0.90 | 0.89, 0.91 | <0.001 |
| Not Insured | 1.37 | 1.34, 1.40 | <0.001 | 1.23 | 1.20, 1.26 | <0.001 |
| Other | 0.84 | 0.81, 0.87 | <0.001 | 0.88 | 0.85, 0.91 | <0.001 |
| Education (% NO HSD) (ref: 15.3% +) |  |  |  |  |  |  |
| 9.1% - 15.2% | 1.01 | 1.00, 1.03 | 0.039 | 1.04 | 1.03, 1.06 | <0.001 |
| 5.0% - 9.0% | 0.97 | 0.96, 0.98 | <0.001 | 1.05 | 1.03, 1.06 | <0.001 |
| < 5.0% | 0.88 | 0.87, 0.90 | <0.001 | 1.02 | 1.00, 1.04 | 0.017 |
| Income (ref: < $46,277) |  |  |  |  |  |  |
| $46,277 - $57,856 | 0.97 | 0.96, 0.99 | <0.001 | 0.98 | 0.96, 0.99 | 0.007 |
| $57,857 - $74,062 | 0.90 | 0.88, 0.91 | <0.001 | 0.91 | 0.90, 0.93 | <0.001 |
| $74,063 + | 0.80 | 0.79, 0.81 | <0.001 | 0.86 | 0.85, 0.88 | <0.001 |
| Facility Type (ref: Non-Academic) |  |  |  |  |  |  |
| Academic | 0.64 | 0.63, 0.65 | <0.001 | 0.79 | 0.78, 0.80 | <0.001 |
| Location (ref: Northeast) |  |  |  |  |  |  |
| Midwest | 1.13 | 1.11, 1.14 | <0.001 | 1.10 | 1.08, 1.11 | <0.001 |
| South | 1.11 | 1.10, 1.13 | <0.001 | 1.04 | 1.02, 1.05 | <0.001 |
| West | 1.02 | 1.00, 1.03 | 0.026 | 1.03 | 1.01, 1.04 | <0.001 |
| MELD (10 Units) | 1.15 | 1.14, 1.16 | <0.001 | 1.11 | 1.09, 1.12 | <0.001 |
| Cirrhosis (No) |  |  |  |  |  |  |
| Yes | 1.10 | 1.07, 1.13 | <0.001 | 1.08 | 1.05, 1.12 | 0.001 |
| AFP (ref: negative) |  |  |  |  |  |  |
| Positive | 1.63 | 1.60, 1.65 | <0.001 | 1.28 | 1.25, 1.31 | <0.001 |
| Treatment (ref: ablation) |  |  |  |  |  |  |
| Resection | 0.78 | 0.76, 0.80 | <0.001 | 0.74 | 0.72, 0.76 | <0.001 |
| Transplant | 0.40 | 0.39, 0.41 | <0.001 | 0.40 | 0.39, 0.41 | <0.001 |
| Non-curative treatment | 2.48 | 2.44, 2.52 | <0.001 | 1.76 | 1.73, 1.79 | <0.001 |

Abbreviations: CI, confidence interval; HR, hazard ratio; AJCC, American Joint Committee on Cancer; HSD, high school diploma; MELD, Model for End-Stage Liver Disease; AFP, alpha-fetoprotein.

Univariable and multivariable Cox proportional hazards regression analyses were performed, and data are presented as hazard ratios (HRs) with 95% confidence intervals (CIs). For this analysis, all cases (alive or death with any follow-up time) were included.

**Supplementary Table 2. Multivariable Logistic Regression as Sensitivity analysis, excluding patients diagnosed after 2011**

| Characteristic | Multivariable | | |
| --- | --- | --- | --- |
|  | **OR** | **95% CI** | **p-value** |
| Age (10 Years) | 0.65 | 0.64, 0.67 | <0.001 |
| Sex (ref: male) |  |  |  |
| Female | 1.34 | 1.27, 1.44 | <0.001 |
| Race / Ethnicity (ref: White) |  |  |  |
| Hispanic | 1,34 | 1.22, 1.46 | <0.001 |
| Black | 0.90 | 0.82, 0.99 | 0.021 |
| Asian + Others | 1.93 | 1.76, 2.11 | <0.001 |
| Tumor Size (cm) | 0.92 | 0.91, 0.93 | <0.001 |
| AJCC T Staging, (ref: T1) |  |  |  |
| T2 | 0.68 | 0.63, 0.72 | <0.001 |
| T3 | 0.31 | 0.29, 0.35 | <0.001 |
| T4 | 0.26 | 0.20, 0.33 | <0.001 |
| AJCC N Staging (ref: N0) |  |  |  |
| N1 | 0.47 | 0.42, 0.59 | <0.001 |
| AJCC M Staging (ref: M0) |  |  |  |
| M1 | 0.32 | 0.25, 0.37 | <0.001 |
| Comorbidity Score (ref:0) |  |  |  |
| 1 | 1.15 | 1.07, 1.23 | <0.001 |
| 2 | 1.01 | 0.92, 1.13 | 0.843 |
| ≥3 | 0.95 | 0.88, 1.03 | 0.228 |
| Insurance (ref: Medicaid/Medicare) |  |  |  |
| Private | 1.66 | 1.56, 1.76 | <0.001 |
| Not Insured | 0.84 | 0.73, 1.00 | 0.033 |
| Other | 1.38 | 1.13, 1.72 | <0.002 |
| Education (% NO HSD) (ref: 15.3% +) |  |  |  |
| 9.1% - 15.2% | 0.90 | 0.83, 0.98 | 0.012 |
| 5.0% - 9.0% | 0.92 | 0.83, 1.00 | 0.058 |
| < 5.0% | 1.00 | 0.91, 1.13 | 0.942 |
| Income (ref: < $46,277) |  |  |  |
| $46,277 - $57,856 | 1.22 | 1.09, 1.31 | 0.007 |
| $57,857 - $74,062 | 1.29 | 1.17, 1.41 | <0.001 |
| $74,063 + | 1.66 | 1.47, 1.80 | <0.001 |
| Facility Type (ref: Non-Academic) |  |  |  |
| Academic | 1.65 | 1.55, 1.76 | <0.001 |
| Location (ref: Northeast) |  |  |  |
| Midwest | 0.84 | 0.77, 0.92 | <0.001 |
| South | 0.92 | 0.85, 0.99 | 0.026 |
| West | 0.98 | 0.90, 1.07 | 0.663 |
| MELD (10 Units) | 0.83 | 0.83, 0.87 | <0.001 |
| Cirrhosis (No) |  |  |  |
| Yes | 0.67 | 0.66, 0.77 | 0.001 |
| AFP (ref: negative) |  |  |  |
| Positive | 0.65 | 0.61, 0.69 | <0.001 |
| Treatment (ref: ablation) |  |  |  |
| Resection | 2.39 | 2.21, 2.70 | <0.001 |
| Transplant | 8.43 | 7.62, 9.19 | <0.001 |
| Non-curative treatment | 0.49 | 0.45, 0.53 | <0.001 |

Abbreviations: CI, confidence interval; OR, odds ratio; AJCC, American Joint Committee on Cancer; HSD, high school diploma; MELD, Model for End-Stage Liver Disease; AFP, alpha-fetoprotein.

Multivariable logistic regression analysis was performed to assess survival as a binary outcome (≥10 years vs. <5 years), and data are presented as odds ratios (ORs) with 95% confidence intervals (CI). In this analysis, cases who died between 5-10 years and cases who were alive with less than 10 years of follow-up were excluded.

**
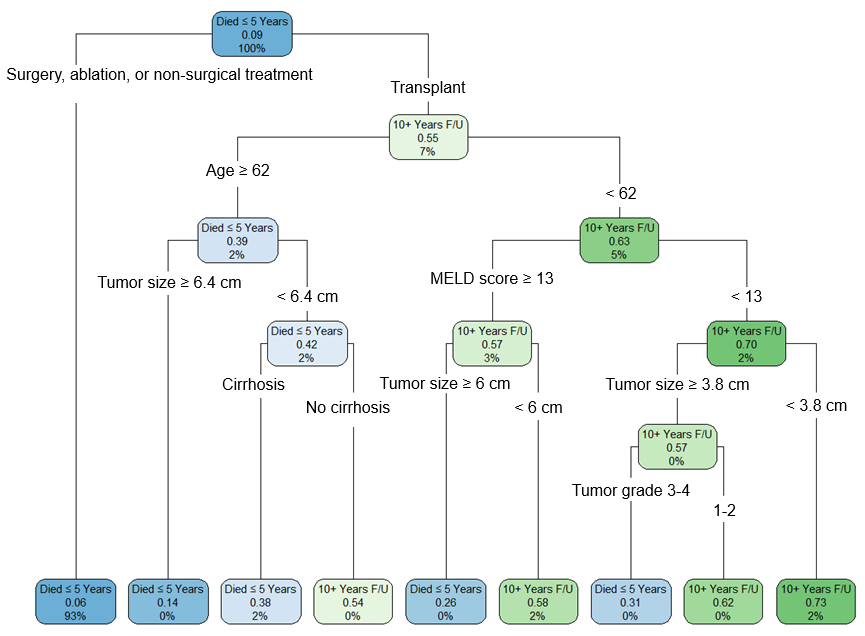
**

**Supplementary Figure 1a.** **Major determinants of ≥10-year survival in patients with hepatocellular carcinoma based on decision tree analysis among patients with T1 tumors.**

Decision tree showing major determinants of ≥10-year survival among patients with T1 hepatocellular carcinoma. In this analysis, cases who died between 5-10 years and cases who were alive with less than 10 years of follow-up were excluded.

Each node reports: the dominant outcome (top), the probability of ≥10-year survival (middle; e.g., 0.55 = 55%), and the proportion of the T1 cohort represented by that node (bottom). Green nodes indicate subgroups with >50% probability of ≥10-year survival; blue nodes indicate subgroups with <50% probability. Overall probability of ≥10-year survival in the T1 cohort was 9% (root node). Liver transplant was associated with markedly higher long-term survival (transplant node: 0.55, 7% of T1 patients). Within transplant recipients, younger age, lower MELD, and smaller tumor size identified subgroups with progressively higher probabilities of ≥10-year survival, with the highest terminal node showing 73% probability (transplant + age <62 + MELD <13 + tumor <3.8 cm; ~2% of the T1 cohort). Non-transplant curative therapies (resection/ablation) remained dominated by early mortality in most branches.

**
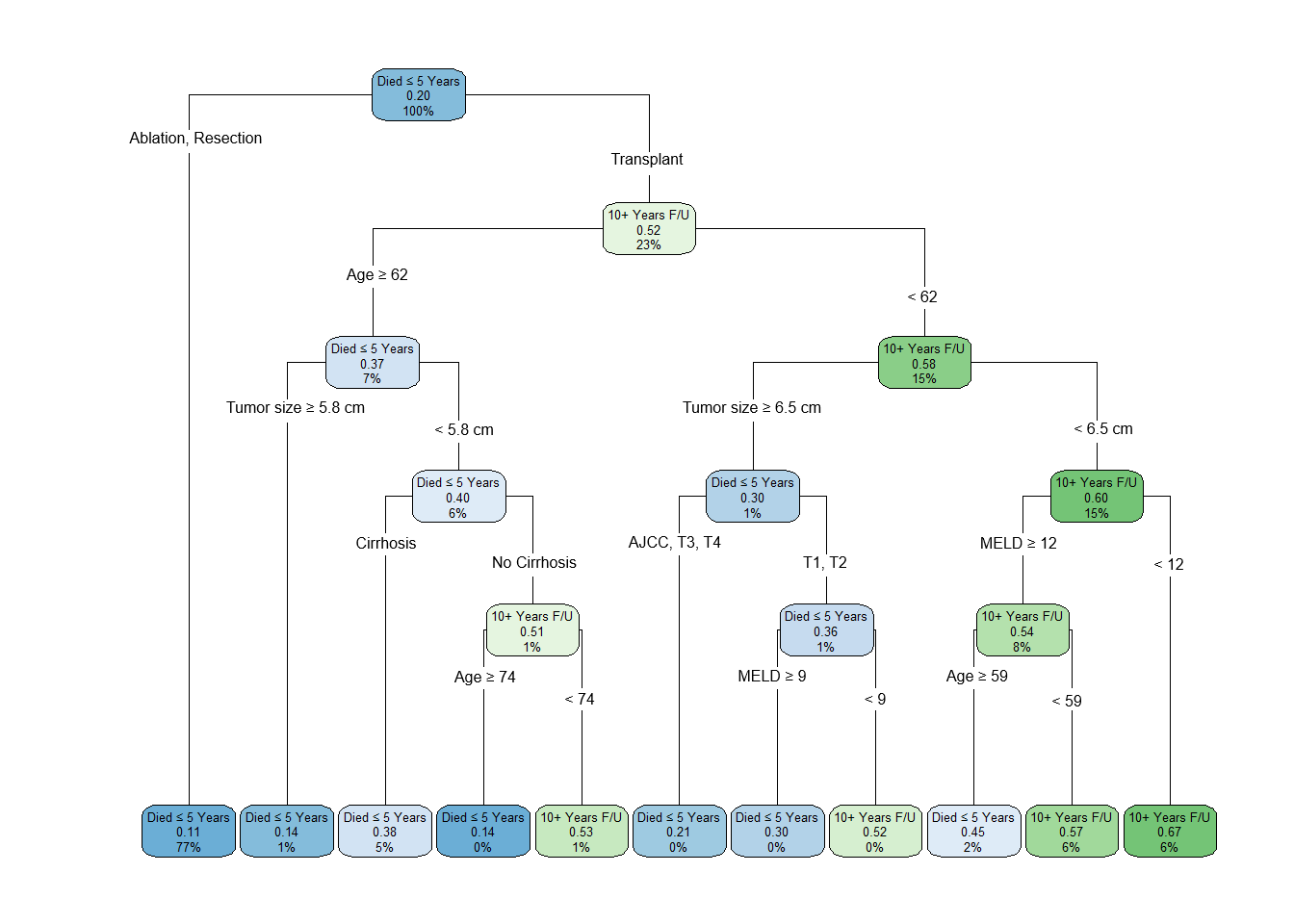
**

**Supplementary Figure 1b.** **Major determinants of ≥10-year survival in patients with hepatocellular carcinoma based on decision tree analysis in patients who underwent curative treatment.**

Decision tree identifying major determinants of ≥10-year survival among patients with hepatocellular carcinoma who underwent curative treatment. In this analysis, cases who died between 5-10 years and cases who were alive with less than 10 years of follow-up were excluded.

Each node shows: dominant outcome (top), probability of ≥10-year survival (middle; e.g., 0.52 = 52%), and proportion of the entire cohort represented by that node (bottom). Green nodes indicate subgroups with >50% probability of ≥10-year survival; blue nodes indicate subgroups with <50% probability. In this cohort LT is associated with substantially higher long-term survival (transplant node: 0.52, 23% of cohort). Within the transplant branch, younger age, smaller tumor size and lower MELD score progressively increase the probability of ≥10-year survival (best terminal node: age <62, tumor <6.5 cm, MELD <12 → 0.67, ~6% of cohort). Non-transplant curative treatments (resection/ablation) remain dominated by early mortality in most branches (blue nodes).
